# Analysis of the Influence of Starting Materials and Processing Conditions on the Properties of W/Cu Alloys

**DOI:** 10.3390/ma10020142

**Published:** 2017-02-08

**Authors:** Isabel Montealegre-Meléndez, Cristina Arévalo, Eva M. Perez-Soriano, Erich Neubauer, Cristina Rubio-Escudero, Michael Kitzmantel

**Affiliations:** 1 Department of Engineering and Materials Science and Transportation, School of Engineering, University of Seville, Camino de los Descubrimientos s/n, 41092 Seville, Spain; carevalo@us.es (C.A.); evamps@us.es (E.M.P.-S.); 2 RHP-Technology GmbH, Forschungs- und Technologiezentrum, 2444 Seibersdorf, Austria; erich.neubauer@rhp-technology.com (E.N.); michael.kitzmantel@rhp-technology.com (M.K.); 3 Department of Computer Languages and Systems, University of Seville, Avenida Reina Mercedes s/n, 41012 Seville, Spain; crubioescudero@us.es

**Keywords:** tungsten-copper alloys, microstructure, Rapid Sinter Pressing (RSP), thermal conductivity

## Abstract

In this work, a study of the influence of the starting materials and the processing time used to develop W/Cu alloys is carried out. Regarding powder metallurgy as a promising fabrication route, the difficulties in producing W/Cu alloys motivated us to investigate the influential factors on the final properties of the most industrially demanding alloys: 85-W/15-Cu, 80-W/20-Cu, and 75-W/25-Cu alloys. Two different tungsten powders with large variation among their particle size—fine (W_f_) and coarse (W_c_) powders—were used for the preparation of W/Cu alloys. Three weight ratios of fine and coarse (W_f_:W_c_) tungsten particles were analyzed. These powders were labelled as “tungsten bimodal powders”. The powder blends were consolidated by rapid sinter pressing (RSP) at 900 °C and 150 MPa, and were thus sintered and compacted simultaneously. The elemental powders and W/Cu alloys were studied by optical microscopy (OM) and scanning electron microscopy (SEM). Thermal conductivity, hardness, and densification were measured. Results showed that the synthesis of W/Cu using bimodal tungsten powders significantly affects the final alloy properties. The higher the tungsten content, the more noticeable the effect of the bimodal powder. The best bimodal W powder was the blend with 10 wt % of fine tungsten particles (10-W_f_:90-W_c_). These specimens present good values of densification and hardness, and higher values of thermal conductivity than other bimodal mixtures.

## 1. Introduction

Tungsten-copper (W/Cu) alloys offer a desirable combination of properties, such as a low coefficient of thermal expansion (CTE) and high melting point from W, and a high thermal conductivity from Cu. They have been developed and used over a wide range of applications, such as high-voltage electrical contacts, heat sinks, and electronic packaging [1,2,3].

From a manufacturing point of view, the preparation of W/Cu alloys presents several difficulties in obtaining a uniform microstructure with high density. Due to large differences in CTE, density, melting point, and the lack of mutual solubility, these alloys are appropriate candidates for manufacture using powder metallurgy (PM) technology. High sintering temperature and long holding time are required in conventional PM methods, causing grain coarsening. This can also result in Cu leaching out from the W skeleton, contributing to Cu segregation [4,5,6,7]. Non-homogenous microstructure and poor behaviour of the final product are common when using traditional PM methods.

The homogenous distribution of W and Cu was recognised as the key issue in obtaining specimens with desirable properties, such as high thermal conductivity, low CTE, and high density [8,9]. However, the main problem has been the development of a uniform skeleton of W after the consolidation process. In particular, the liquid sintering state of the Cu particles and the infiltration of the Cu in such skeletons is affected by the homogenous distribution of the holes in the W skeleton [10,11,12,13,14].

It has been widely established that the powder selection of the starting materials can contribute to enhancing the final properties of the W/Cu products [15,16,17].

Regarding that, in the present study, the 85-W/15-Cu, 80-W/20-Cu, and 75-W/25-Cu alloys have been synthesized from fine copper particles and several tungsten blends made from two types of W powders, which presented a relevant difference between their particle sizes. In this context, the use of such tungsten blends could contribute to improve the uniformity of the alloys’ microstructures and to enhance their properties, such as hardness and densification.

With the goal of guaranteeing the reduction of the holding time, rapid sinter pressing (RSP) has been presented as a novel technique to fabricate W/Cu specimens. The benefit of this manufacturing method has been proven by the short time employed to produce dense and compact specimens [18].

This paper aims to simplify the manufacturing process of W/Cu alloys (85-W/15-Cu, 80-W/20-Cu, and 75-W/25-Cu) from fine Cu and W bimodal powder combinations under several processing conditions to create W/Cu specimens with homogeneous microstructures and enhanced densification, hardness, and thermal conductivity.

## 2. Materials and Methods

### 2.1. Materials

The selection of Cu powder was based on previous research, which recommended the use of fine Cu particles to synthesize W/Cu alloys with suitable properties [10,18]. This Cu powder was produced by a special classifying process of powder generation with median (d_50_) particle size (<20 µm). Two types of W powders were chosen according to their average particle size: a fine (W_f_) and a coarse (W_c_) powder, supplied by Wolfram Bergbau & Hütten AG.

**Figure 1:**
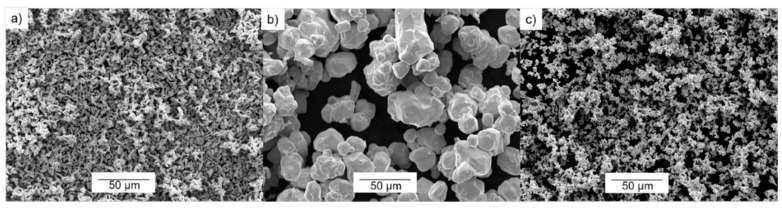
Secondary Electron-SEM images of the starting powders: (**a**) Cu powder; (**b**) Coarse W powder (W_c_); (**c**) Fine W powder (W_f_).

A characterization of the starting powders was performed, verifying the information about their morphology and particle size supplied by the manufacturers. Particle size distribution for the starting powders was measured by laser diffraction analysis (Mastersizer 2000, Malvern, Worcestershire, UK), resulting in a median (d_50_) of 5.45 µm and 15.39 µm of d_90_ for Cu powder. The d_50_ particle sizes of the fine W (W_f_) powder and the coarse powder (W_c_) were 8.45 µm and 30.73 µm, respectively. Additionally, the d_90_ particle sizes were 26.50 µm and 69.05 µm, respectively. A morphological study of the powders was carried out. All the scanning electron microscopy (SEM) images of these powders were taken by a JEOL 6460LV microscope (Tokyo, Japan), equipped with an energy dispersive X-ray spectroscopy (EDS) detector. Their morphologies are shown in Figure 1. The Cu powder presents the finest particles (see Figure 1a). Concerning the W powders, both show a faceted morphology (see Figure 1b,c). In the case of W_f_, some particle agglomeration is observed.

### 2.2. Methods

Three weight ratios (%) of fine and coarse W particles (W_f_:W_c_) were evaluated in order to study the effect of W particle size on the specimens’ behavior [19]. These powder blends were named “bimodal powders”. Three W/Cu alloys 85-W/15-Cu, 80-W/20-Cu, and 75-W/25-Cu were produced from the Cu powder and bimodal powder blending (see in Table 1).

**Figure 2:**
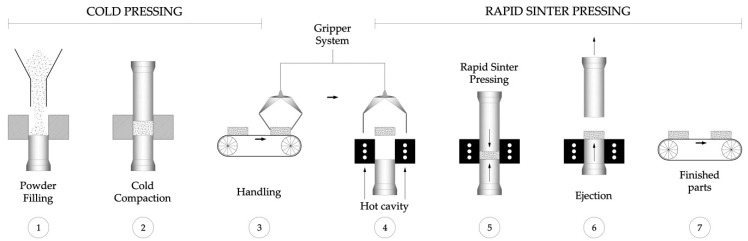
Scheme of the cold consolidation and the rapid sinter pressing processing of the W-Cu alloys.

Blending was performed in a Sintris mixer for 1 h. Then, the powders were compacted by RSP (see Table 2). A self-made hot pressing machine was utilized (rapid sinter pressing equipment of RHP-Technology GmbH & Co. KG, Seibersdorf, Austria). The operation of the RSP device—which uses a permanently heated steel tool—is illustrated in Figure 2.

The specimens—which were precompacted in an automated cold press at 170 MPa for 10 s (see Figure 2, step 2)—were placed into the hot die (see Figure 2, step 4). To avoid reaction between the die and the specimens, a protective coating of boron nitride (BN) was applied to the specimens’ surface. Before the hot compaction, there was a degasification of the full die. The time of this degasification stage was also considered as a processing parameter to be studied (5 or 10 s). The hot consolidation process was performed at 900 °C and 150 MPa sintered/compacted simultaneously at 15 bar/s for 30 s, 60 s, and 90 s.

After hot consolidation, sintered samples were ejected and characterized. Table 2 shows the different time parameters which were tested in the experiments. Regarding the process temperature, there was a technical limitation in the equipment; deformation was observed on the specimens at higher temperatures.

### 2.3. Characterization of the Specimens

To study the microstructure of the specimens, metallographic preparation for all samples was performed. Optical microscopy (OM, Nikon Model Epiphot 200, Tokyo, Japan) and SEM (Jeol 4640) were used to study the morphology of the specimens’ microstructure. Furthermore, the elemental compositions of the specimens were examined by EDS. Densities of the specimens were determined by Archimedes´ method, and three tests were carried out for each sample. Hardness measurements were conducted on polished surfaces using a Vickers hardness tester (HV2-Struers Duramin-A300, 2 kg applied load); five values were obtained for each material. The thermal conductivity (λ) of several specimens was calculated using the estimated specific heat capacity (C_p_), the measured thermal diffusivity (α), and the measured density (ρ) of the specimens, as follows [20]:

λ = α·ρ·C_p_ (1)

using known specific heat capacities for pure W and Cu, being C_p_(W) = 0.134 kJ/kg·K and C_p_(Cu) = 0.386 kJ/kg·K [21]. The thermal diffusivities were obtained by the laser flash method at room temperature (LaserJet (LFA1250/1600)), employing cylindrical samples with a diameter of 20 mm; three measurements were made for each sample.

## 3. Results and Discussion

### 3.1. Microstructure Characteristics

As described by previous authors in liquid phase sintering processes, copper powder began to melt during the manufacturing. Due to the capillary force and the action of surface tension, the liquid copper phase coated the surface of the tungsten particles. If copper cannot effectively fill the gaps among tungsten particles, blind holes are formed [22]. In the present work, the temperature was raised up to 900 °C for 30, 60, or 90 s in order to obtain solid-phase sintering. Therefore, the fluid copper could not fill the pores of the tungsten skeleton.

If a good dispersion could be achieved between copper and tungsten particles, the consolidated specimens would exhibit excellent thermal conductivity properties due to the formed copper network. If not, undesirable empty zones may appear between tungsten particles where copper is not present.

From this point of view, and in order to achieve a correct dispersion, a bimodal tungsten powder is used to avoid lack of copper between large W particles. Homogeneously-distributed small and large particles can contribute to obtaining uniform microstructures in the W/Cu alloys.

**Figure 3:**
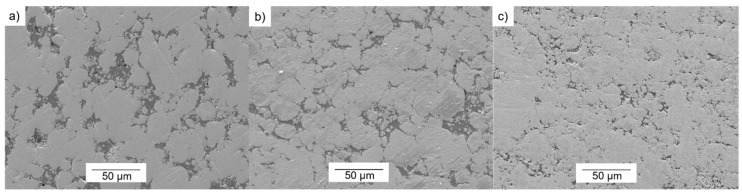
Secondary Electron-SEM images of 85-W/15-Cu alloys prepared from three powder mixings: (**a**) 10-W_f_:90-W_c_; (**b**) 20-W_f_:80-W_c_; (**c**) 30-W_f_:70-W_c_.

Firstly, the microstructures have been considered according to the weight percentage (wt %) of copper. Concerning the microstructure of the 85-W/15-Cu alloys, the influence of the fine tungsten powder (W_f_) is more significant than in the other alloys, due to the large amount of tungsten that this alloy presents. In Figure 3, the variations of the particle size of the tungsten powder can be seen for the microstructures obtained from 85-W/15-Cu alloys made from the three W bimodal powders under the same manufacturing conditions (900 °C, 150 MPa, 10 s for degasification and 90 s for hot consolidation time).

In the microstructures shown in Figure 3, slight agglomerations of the fine tungsten particles (W_f_) could be generally observed. This phenomenon is more pronounced in the 85-W/15-Cu alloy with tungsten powder that has been prepared from 30-W_f_:70-W_c_ (see Figure 3c). This appreciation is based on optical and electron microscopy images. In Figure 3a,b, small and isolated particles of tungsten could be identified separated from the coarse ones. These agglomerations occurred in spite of the previously optimized mixing stage; a more in-depth study could be performed to study the mixing effect.

Furthermore, the microstructure images reveal that agglomerations of fine W particles could affect the behavior of the specimens, reducing their densification. Squeezing and contact between tungsten particles could cause blind holes that copper cannot effectively fill. This is consistent with literature that reports that the formation of blind holes decreases as the copper content increases [22]. Therefore, the alloy with 85 wt % of W could have a higher number of blind holes than the other alloys with 80 wt % and 75 wt % of tungsten. Among 85-W/15-Cu alloys, the specimens from bimodal powders of 10-W_f_:90-W_c_ and 20-W_f_:80-W_c_ showed less closed voids (see Figure 3).

**Figure 4:**
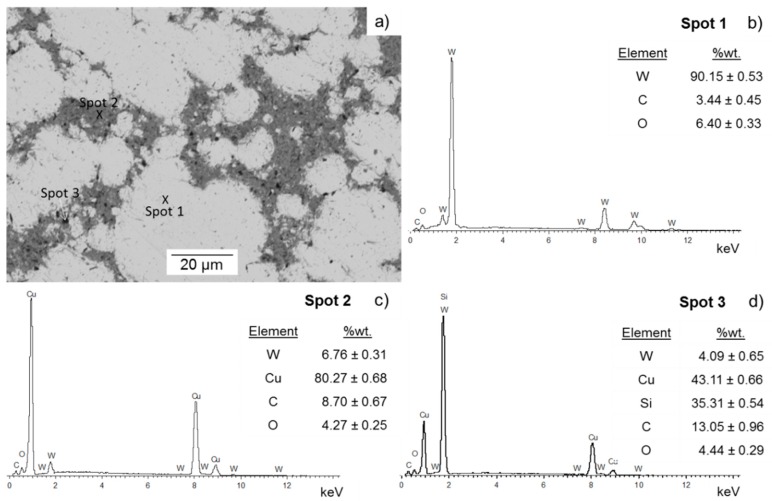
(**a**) Back Scattered Electron-SEM image of 85-W/15-Cu alloy from W bimodal powder 10-W_f_:90-W_c_ powder produced under 10 s for degasification and 90 s for hot consolidation; (**b**) energy dispersive X-ray spectroscopy (EDS) spectrum in Spot 1 (W phase); (**c**) EDS spectrum in Spot 2 (Cu phase) and (**d**) EDS spectrum in Spot 3 (impurities from metallographic preparation).

Figure 4 reveals the microstructure of the 85-W/15-Cu alloy from the bimodal powder with the highest content of coarse tungsten particles (10-W_f_:90-W_c_). The light grey and dark grey colors indicate W and Cu phases, respectively. EDS analysis in Spot 1 and Spot 2 illustrate this in Figure 4b,c, respectively. Additional impurities from the metallographic preparation are detected as observed in Spot 3 (see Figure 4d). In Figure 4, a uniform distribution of Cu is not observed between the large areas of W. However, slight connectivity between the Cu regions can be seen. Moreover, no residual porosity can be easily identified in Figure 4a. This specimen was manufactured under 10 s of degasification time and hot pressed for 90 s. From the perspective of the processing route, these parameters may be the best consolidation times to achieve the lowest porosity, and therefore the maximum densification.

**Figure 5:**
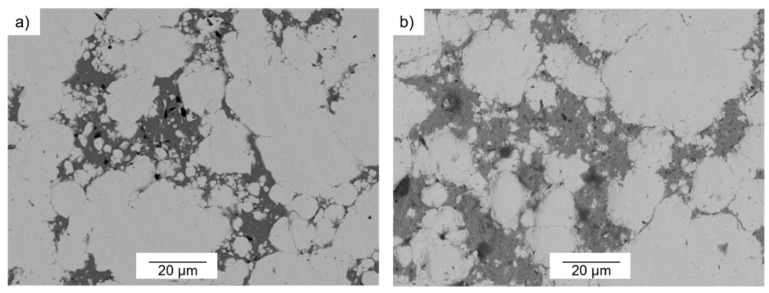
(**a**) Back Scattered Electron-SEM image of 80-W/20-Cu alloys prepared from bimodal powder 10-W_f_:90-W_c_, degasification time 10 s and hot consolidation time 90 s; (**b**) Back Scattered Electron-SEM image of 85-W/15-Cu alloys prepared from bimodal powder 10-W_f_:90-W_c_, degasification time 10 s and hot consolidation time 30 s.

The microstructures of the specimens from 80-W/20-Cu alloys showed good homogeneous distribution of the fine tungsten particles (W_f_). Figure 5a shows the microstructure of this alloy from the bimodal powder 10-W_f_:90-W_c_ processed with 10 s degasification and 90 s hot consolidation.

Observing the effect of processing time on the microstructure of the specimens, short processing time could cause small pores in the copper region independently of the copper content, as shown in Figure 5b.

Comparing the SEM-images in Figure 4a and Figure 5a, there are slight differences apart from the Cu content. The higher the Cu content, the lower the agglomeration between the fine tungsten particles (W_f_) and, therefore, the better the uniform microstructure of the specimens. At lower fine tungsten powder content, less agglomeration between particles occurs, as could be expected. However, the lowest amount of fine tungsten powder (10 wt %) enabled the enhancement of the densification. This is due to the fact that fine tungsten particles are able to enter into the pores of the W skeleton by the applied pressure during the pressing–sintering process [22].

**Figure 6:**
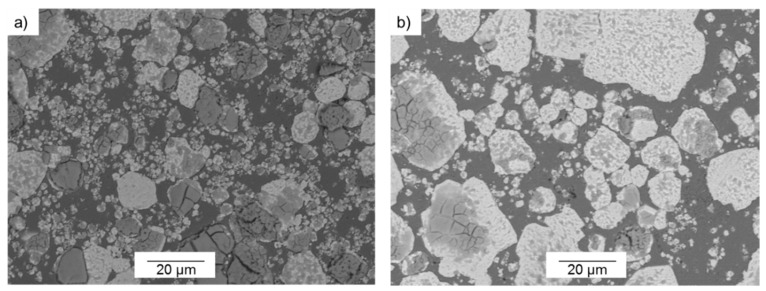
(**a**) Back Scattered Electron-SEM image of 75-W/25-Cu alloy prepared from bimodal powder 30-W_f_:70-W_c_, degasification time 5 s, and hot consolidation time 90 s; (**b**) Back Scattered Electron-SEM image of 75-W/25-Cu alloy prepared from bimodal powder 10-W_f_:90-W_c_, degasification time 10 s and hot consolidation time 90 s.

A comparison between the microstructures of the three alloys produced at identical processing conditions (85-W/15-Cu, 80-W/20-Cu, and 75-W/25-Cu) can be done by studying the microstructures in Figure 4a, Figure 5a, and Figure 6b. Increasing the copper content up to 25 wt %. Leads to the formation of contacts between the copper areas, as can be seen in Figure 6b. This is desirable to enhance the thermal conductivities of the alloys.

As previously mentioned, the higher the copper content, the smaller the agglomeration of the fine tungsten particles. In spite of the 30 wt % content of fine tungsten particles, the copper phase formed during the hot consolidation stage contributes to the dispersal of the fine tungsten particles (see Figure 6a). The blind holes were not observed, due to the homogenous distribution of the tungsten particles. These results agree with the literature reports [22].

The degasification stage is necessary to exclude any moisture and discharge any adsorbed gases on the particles in solid stage. However, there is no influence of the degasification times (5 s and 10 s) on the microstructures of specimens (see Figure 6a,b). This can be attributed to the fact that both degasification times were too short to detect possible variations in the specimens’ microstructures.

### 3.2. Densification and Hardness

**Figure 7:**
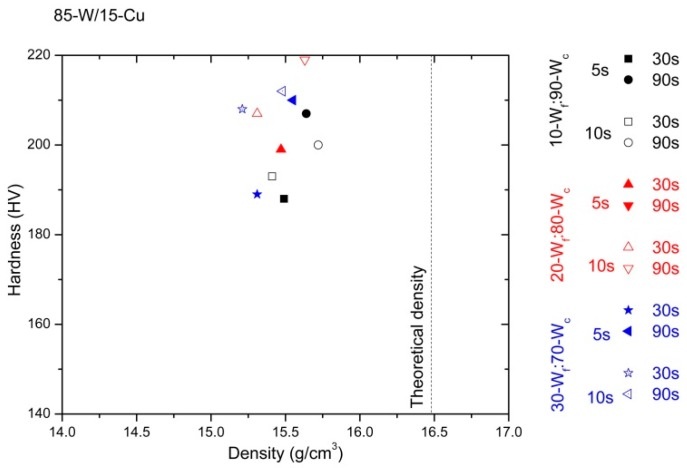
Hardness vs. density of 85-W/15-Cu alloy prepared from three different starting powder mixes and produced under different processing times: degasification times of 5 s and 10 s, in addition to consolidation times of 30 s and 90 s.

**Figure 8:**
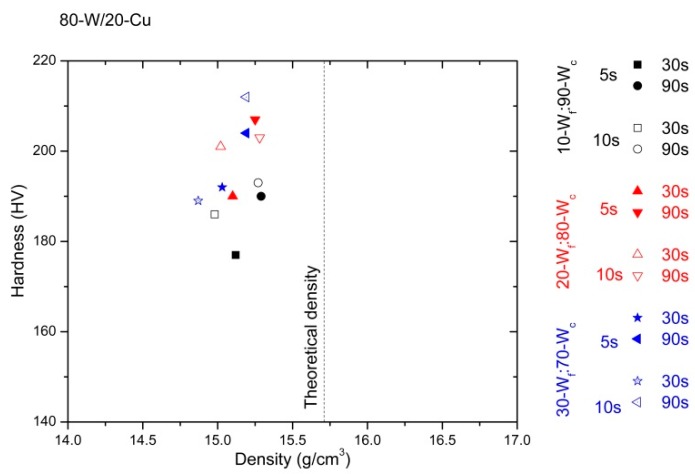
Hardness vs. density of 80-W/20-Cu alloy prepared from three different starting powder mixes and produced under different processing times: degasification times of 5 s and 10 s, in addition to consolidation times of 30 s and 90 s.

**Figure 9:**
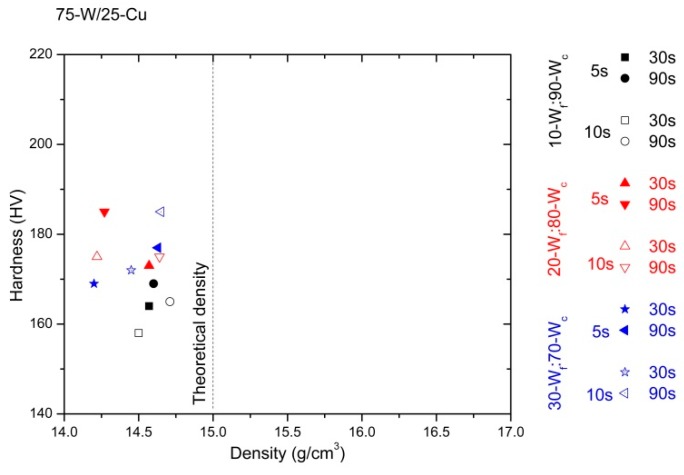
Hardness vs. density of 75-W/25-Cu alloy prepared from three different starting powder mixes and produced under different processing times: degasification times of 5 s and 10 s, in addition to consolidation times of 30 s and 90 s.

The values of the density and hardness measurements are divided considering the content of copper (wt %) of the specimens. These results are represented in Figure 7, Figure 8 and Figure 9. In order to simplify the presentation of the data, the corresponding values for 60 s have not been included; they do not contribute to the discussion of the results. Theoretical densities are obtained from the rule of mixing for every alloy.

Comparing the densification of the three alloys (85-W/15-Cu, 80-W/20-Cu, and 75-W/25-Cu), the higher the copper content, the higher the densification of the specimens. This is consistent with the literature [23,24]. Furthermore, the hardness of the specimens displays the opposite trend; the higher the tungsten content (wt %), the higher the hardness that the specimens present.

The processing parameters strongly influence the behavior of the specimens, independently of the type of alloy. The effect of their variations can be seen in the values of densification and hardness. It is also verified that the increment of the hot consolidation time drives improvements in the densification, independently of the degasification time (5 s or 10 s); 90 s reaches the best values.

Regarding the bimodal tungsten powder, the alloys made from 30-W_f_:70-W_c_ generally offer lower properties than alloys made from 10-W_f_:90-W_c_ powder. The effect of the agglomeration of fine particles may contribute to this decrement in specimens’ densification. However, these are better results than in alloys made from one type of W powder under similar conditions [18].

The range of densification values for each alloy is presented in the following. In samples from 85-W/15-Cu, this range varies between 92.6% and 95.7%. These values are obtained with the use of 30-W_f_:70-W_c_ (degasification for 10 s and hot consolidation for 30 s) and 10-W_f_:90-W_c_ (degasification for 10 s and hot consolidation for 90 s).

The results of density and hardness in 80-W/20-Cu alloy are shown in Figure 8. The densification range oscillates from 94.67% to 97.34% in specimens with 30-W_f_:70-W_c_ (degasification for 10 s and hot consolidation for 30 s) and 10-W_f_:90-W_c_ (degasification for 5 s and hot consolidation for 90 s), respectively.

Even the lowest bimodal value obtained at 900 °C is higher than the best relative density measured in specimens produced with one type of coarse W powder under the same processing method, except at 950 °C, hot consolidation for 80 s, and degasification for 5 s (92.7%). This means that the use of bimodal powder can contribute to reduce the processing temperature below 900 °C and to enhance the densification of the specimens.

The densities and hardness of the 75-W/25-Cu alloy are shown in Figure 9. The relative density of the alloy made from 30-W_f_:70-W_c_ (degasification for 10 s and hot consolidation for 30 s) and 10-W_f_:90-W_c_ (degasification for 10 s and hot consolidation for 90 s), increased from 94.9% to 98.4%.

Moreover, a comparison between the graphs shows that under this procedure, the 80-W/20-Cu alloys manufactured present the more controlled densification values (narrower distribution, Figure 8).

Comparing hardness values, specimens from monomodal W powders under the same processing method (at 950 °C, hot consolidation for 80 s, and degasification for 5 s) display dispersion between 155 up to 200 HV [18]; specimens from bimodal W powders from 177 to 212 HV (Figure 8). Therefore, the use of W bimodal powder at low processing temperature leads to the achievement of specimens with a suitable behavior.

### 3.3. Thermal Conductivity

The measurement of the thermal conductivity was performed only in specimens hot consolidated for 90 s, due to their good densification and hardness values. Furthermore, the alloys considered for the determination of their thermal conductivity were 85-W/15-Cu and 80-W/20-Cu, because of their industrial interest. Moreover, these two types of alloys may be more affected by poor contiguity of the copper particles.

**Figure 10:**
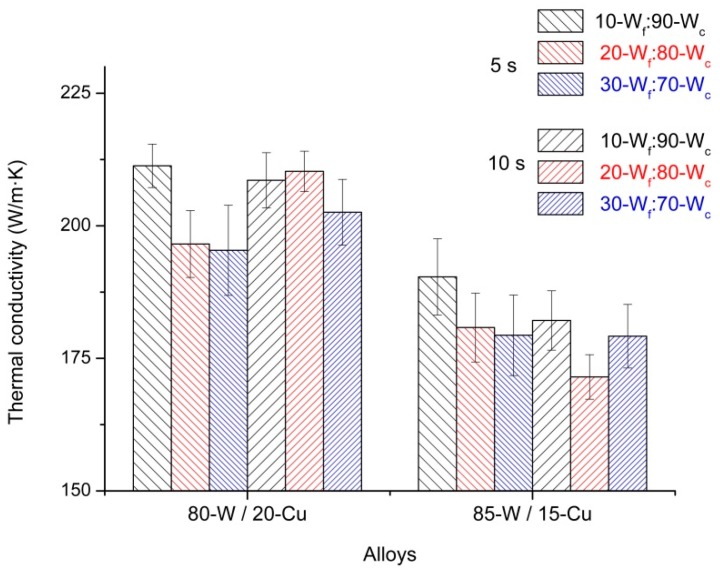
Thermal conductivity of the specimens produced from the three bimodal powders hot consolidated for 90 s.

Thermal conductivity data are in agreement with the microstructure, densification, and hardness results described above, as seen in Figure 10. The alloys with the highest content of fine tungsten particles present the lowest values of thermal conductivity (30-W_f_:70-W_c_). This is related to the agglomeration of the particles in addition to the closed porosity observed in Figure 5.

However, despite this issue, the advantage of using W bimodal powders is evident. In measured specimens of 80-W/20-Cu alloy with different bimodal powders, the thermal conductivity values rise up to 211 W/m·K. In a previous work with monomodal W powder, the maximum value measured for this type of alloy was 176 W/m·K [18] in specimens hot consolidated at 950 °C for 80 s and degasification time of 5 s. In the literature, the 80-W/20-Cu alloy made from Cu-coated W powder showed thermal conductivity values of 146 W/m·K [12].

## 4. Conclusions

Several main conclusions can be drawn from this research. Regarding the raw materials processed at the same parameters by RSP, the microstructure of the specimens was directly influenced by the copper content. The 80-W/20-Cu alloys showed more homogeneous microstructure than 85-W/15-Cu and 75-W/25-Cu alloys.

The higher the content of fine tungsten particles (W_f_) in the bimodal powder, the larger the observed agglomeration. The best samples produced by bimodal W powders were made with 10 wt % of fine tungsten particles (10-W_f_:90-W_c_). These specimens present better values of densification and hardness, and higher values of thermal conductivity than other bimodal mixtures, considering the estimated average values.

From a manufacturing point of view for the W/Cu alloys, 90 s is considered the best sinter/consolidation time, achieving better values of densification and hardness than 60 s and 30 s.

The degasification time effect is not clearly identified in the final behaviour of the alloys.

Compared to monomodal alloys, an appropriate selection of bimodal mixture can lead to an improvement in the final properties, even decreasing the processing temperature.

## Figures and Tables

**Table 1:** Different weight ratios (wt %) of the W bimodal powder combinations (W_f_:W_c_) for the produced W/Cu alloys.

Alloy	85-W/15-Cu			80-W/20-Cu			75-W/25-Cu		
W_f_:W_c_ (%)	10:90	20:80	30:70	10:90	20:80	30:70	10:90	20:80	30:70

**Table 2:** Rapid sinter pressing (RSP) time processing parameters tested.

Degasification Time (s)	Hot Consolidation Time (s)
5, 10	30, 60, 90
